# Sero-prevalence of malaria and the knowledge, attitudes and practices relating to the prevention of malaria among indigenous people living in the central forest spine in Peninsular Malaysia: a mixed-methods study

**DOI:** 10.1186/s12936-022-04293-5

**Published:** 2022-10-03

**Authors:** Siti Fatimah Kader Maideen, Abdul Rashid, Nur Indah Ahmad, Siti Nur Afiqah Zahari, Rukman Awang Hamat

**Affiliations:** 1 Department of Public Health Medicine, RCSI and UCD Malaysia Campus, Penang, Malaysia; 2 Department of Veterinary Pathology & Microbiology, Faculty of Veterinary Medicine, University Putra Malaysia, Serdang, Malaysia; 3 Department of Microbiology, Faculty of Medicine and Health Sciences, Universiti Putra Malaysia, Serdang, Malaysia

**Keywords:** Prevalence, Malaria, Knowledge, Attitude, Practice, Indigenous populations

## Abstract

**Background:**

Malaria is still a major public health threat in some parts of the world. Many countries are targeting to achieve malaria free status country. This study aimed to determine the sero-prevalence of malaria and the knowledge, attitudes and practices relating to the prevention of malaria among the indigenous adults living in the central forest spine in Peninsular Malaysia.

**Methods:**

A mixed method study was conducted in indigenous settlements in 2020. Blood film for malaria parasite (BFMP) was used to diagnose malaria in this study. A structured questionnaire was used to collect data from the participants. For the qualitative data, in-depth interviews were conducted and data was collected until data saturation was reached. Multiple linear regression was used to determine the predictors after adjusting for confounders. A p-value of < 0.05 is considered as statistically significant. Meaningful statements from the in-depth interviews were assigned to the relevant codes using NVivo version 12 software.

**Results:**

A total of 284 indigenous people participated in the study. The prevalence of malaria in this study was 0%. Those in the middle age group between 25 and 41 years and tested positive for malaria previously were significantly more likely to have better knowledge and attitude scores. Significant correlations were also observed between knowledge-attitude and knowledge-practice. For the qualitative results, most of the respondents were unsure of monkey malaria, but all were aware of human malaria.

**Conclusion:**

The present study highlighted the absence of malaria in the study population and relatively good knowledge, attitudes and practices relating to the prevention of malaria.

## Background

Despite a steady decline in the annual malaria incidence and mortality within the South-East Asia Region, several countries are still faced with challenges brought about by the complexity of the disease before they are able to achieve the aim of malaria-free region by 2030 [1, 2]. The region has the second highest global burden of malaria based on mortality and morbidity and contributes to 58% global burden of *Plasmodium vivax*.

Malaysia was one of the countries within the region targeted to achieve and maintain a malaria-free status country by 2020 [1]. Continuous efforts to alleviate malaria infection since the 1960s has shown progress where the country had entered the elimination phase of indigenous malaria at the end of 2018 after the country reported zero case of human malaria in that year. Preparedness and response to malaria outbreaks, early detection and prompt treatment and community and social mobilization are three of the strategies aligned in the National Strategic Plan for Elimination of Malaria (NSPEM) 2011–2020.

Between 2008 and 2017, Malaysia recorded a reduction in malaria cases by 98.6% [3]. At the same time, improvement in diagnostic capacity by PCR helped to identify the fifth *Plasmodium* species causing malaria in humans, *Plasmodium knowlesi* [4]. *Plasmodium knowlesi* is an emerging zoonotic malaria parasite circulating within wild monkey populations and is transmitted to humans through the bites of *Anopheles* mosquitoes. *Plasmodium knowlesi* causes severe and fatal malaria [2]. Those aged 45 years and above and women have increased risk of deaths. A systematic review and meta-analysis showed pooled prevalence of severe *P. knowlesi* infection of 19% with most of them manifested symptoms, such as kidney injuries, jaundice and hyperparasitaemia [5] and respiratory distress [2]. Malaria is common in East Peninsular Malaysia, with *P. knowlesi* being the common infection [6]. In 2017, the incidence of *P. knowlesi* in East Malaysia was 5.9 per 100,000 population [7]. The case fatality rate for *P. knowlesi* was 2.5 per 1000 population [2]. Forest-related activities, agriculture and plantation activities associated with *P. knowlesi* infection [3].

Communities residing in remote areas near the fringe of forests and socio-economically disadvantaged groups including Indigenous peoples may be more at risk of being infected with malaria due to inaccessibility to immediate diagnostic or healthcare, lack of resources to purchase preventive products such as insecticides, and increased of exposure to vectors due to economic activities which requiring them to be outdoor in the forest [8, 9]. The indigenous people of peninsular Malaysia, locally known as Orang Asli comprise of 3 main groups known as Negrito, Senoi and Proto Malay, of which are then further classified into 18 sub-ethnic groups each with their own unique language and culture [10]. Malaria has frequently been reported prevalent among the Orang Asli living in the states of Peninsular Malaysia [11–13]. Moreover, common preventative measures such as insecticide-treated nets and indoor spraying were shown to be insufficient to protect them as they are exposed to the mosquitoes when they outside their houses [14].

Better knowledge, attitudes and practices related to malaria may help the community against malaria, provided they are given access to resources and services that enable them to put into action. A study among an Orang Asli community in eastern Peninsular Malaysia suggested more health promotion activities to empower and enhance better attitudes and practices associated to managing malaria infections were needed [15]. Malaysia is a tropical country with hot and humid climate, and is currently undergoing rapid changes in the environmental landscapes including intensive use of land for agriculture and urbanisation, which may lead to efficient breeding grounds for mosquitoes to thrive. Several mosquito-borne diseases apart from malaria are also commonly reported in Malaysia, including dengue and Chikungunya. This may cause confusion about the basic understanding about the symptoms of those diseases among the public, especially in communities with poor socio-economic and education levels [16], which may delay them from getting appropriate medical treatment. Although studies have been conducted on this theme in parts of Malaysia, the differences in the way the people live and in their beliefs in the many indigenous communities means more in-depth information is needed to support the achievement of the goal of complete malaria eradication in Malaysia.

This study aims to understand the level of knowledge, attitudes and practices related to malaria among three ethnic groups of Orang Asli, the Semai, Temiar and Jahai living in the central spine forest of peninsular Malaysia. These communities live by the fringe or within forested areas and often engage in hunting, gathering, and small-scale farming. This, along with limited access to medical care and preventative measures against malaria, places them at high risk of being bitten by mosquitoes [14, 17, 18]. Findings from this study will provide valuable information to develop focused strategies to prevent and detect early signs of malaria and subsequently achieve the objective of the National Strategic Plan for Elimination of Malaria 2011–2020.

## Methods

### Study design

This mixed methods (quantitative and qualitative) study was conducted in 2020.

### Setting and study population

This study was carried out in nine Indigenous settlements located along the central spine forest range in Peninsular Malaysia. The exact number of villagers fluctuate because many still practice nomadic lifestyles and because some are hunter and gatherers, they may be away from the village for days hunting and foraging for food in the forest. Women are also involved in the plantation and agricultural activities.

### Sampling

All the villagers aged 18 years old and above were eligible to participate in the study. A non-purposive sampling method was chosen because there was no sample frame of the villagers because the number of villagers in the village may fluctuate at any one time. For the qualitative data, in-depth interviews were conducted and data was collected until saturation was reached. A non-purposive sampling method was also used to recruit participants for the qualitative component. Participants were chosen based on their talkative nature and willingness to be interviewed in depth. Thirteen participants were interviewed in-depth.

The respondents' socio-demographic characteristics are shown in Table 1. Their ages ranged from 20 to 43 years old (mean 30.5). The majority of participants (10) were women, married with children. Except for one participant who was unemployed, twelve of them worked full-time. The majority (eight) had completed secondary school and stayed with their partners. Their monthly income ranged from RM 0 to RM1500 (mean 747.70) (USD1 = RM4).

**Table 1:** Socio-demographic characteristics of the qualitative participants

Respondents	Age (year)	Marital status	Education level	Job	Type of job	Monthly income	Living arrangement
R1 (Male)	32	Married with children	Secondary	Full time	Farmer	RM1000	Partner
R2 (Female)	33	Married with children	Secondary	Full time	Housewife	RM1500	Partner
R3 (Female)	43	Married with children	Primary	Full time	Housewife	RM900	Partner
R4 (Female)	29	Married with children	Secondary	Full-time	Housewife	RM900	Partner
R5 (Female)	20	Single	Secondary	Not working	NA	NA	Family
R6 (Female)	42	Married with children	Secondary	Full Time	Housewife	RM1500	Partner
R7 (Female)	31	Married with children	Secondary	Full time	Housewife	RM1000	Partner
R8 (Female)	27	Married with children	Primary	Full time	Housewife	RM1000	Partner
R9 (Female)	23	Married	Secondary	Full time	Housewife	RM400	Partner
R10 (Female)	25	Married with children	Secondary	Full time	Personal shopper	RM400	Family
R11 (Male)	38	Widowed with children	Primary	Full time	Rubber Tapper	RM500	Children
R12 (Male)	27	Married with children	Primary	Full time	Rubber Tapper	RM300	Partner
R13 (Female)	26	Married with children	Illiterate	Full time	Housewife	RM320	Partner

### Tool

A questionnaire specially designed for this study was used. The interviews were conducted in the participant’s homes by trained investigators using a uniform protocol which was set up to minimize error and bias. Besides the baseline demographic information which consisted of sex, age, race, marital status, education level, employment status and income; information was also collected concerning the knowledge, attitudes and practices related to malaria. Information was also collected on the history of malaria infection and history of being tested for malaria.. The questions on knowledge included if the participants were aware of malaria, the signs and symptoms, natural history, prognosis, its transmission and prevention. Questions on attitude included health seeking behaviours and on prevention. Questions on practice including practices related to prevention. For the qualitative component of the study, questions included on the awareness of malaria, transmission, treatment and its prevention. In-depth interviews were conducted using a semi-structured interview guide. The data was collected using face to face interviews in the Malay language. After interviewing 13 participants, data saturation was achieved.

Thin and thick blood films were prepared on the same glass slide. Both films were completely air dried for approximately 30 min, then absolute methanol was used to fix the thin blood film. The blood films were stained with 3% Giemsa for 45 min according to the established protocols [19]. The slides were carefully examined in a blinded method by two independent-trained microscopists. The slides viewing were headed by a Professor of Medical Microbiology and a Consultant Clinical Microbiologist. Quality check of the slides were done by other department members in the faculty.

### Analysis

Each correct answer for knowledge, attitude and practices was given one mark each and no marks were given for wrong answers. Higher cumulative marks suggested better knowledge, attitude and practice. Data was analysed using IBM Statistics SPSS Software version 23. Descriptive data is presented in tables. T-test and ANOVA were used to compare means of the knowledge, attitude and practice scores with age, sex, marital status, education and monthly household income, and ‘know someone infected with malaria’ and history of having tested for malaria for statistical significance. Linear regression was conducted to factor in confounders. A probability value of p < 0.05 was considered as significant. Qualitative: The verbal data obtained from the in-depth interviews were transcribed verbatim. Before coming out with the codes and themes, repeated reading was performed by the researchers to familiarise with the contents. The researchers re-read the transcripts and identified codes after looking at the patterns of data, taking important notes and marking ideas for the coding process. The coding framework identified mirrored the idea of knowledge, attitude and practice in preventing malaria. Meaningful statements were assigned to the relevant codes using NVivo version 12 software. The codes were sorted into potential themes and sub-themes and this step was established after a consensus through discussion among the research team members. The extracted data were reviewed to determine their relevance and relationship with the themes or sub-themes.

### Ethics

This study received the approval from the Joint Penang Ethical Committee (JPEC 20-0020) and the department in charge of the welfare of the indigenous people. The participant information sheet was read out to the respondents and an informed verbal consent was obtained before commencing. The respondents had the right to refuse and exit from the study at any point of the research.

## Results

A total of 284 Orang Asli participated in the study. Majority of the participants were women (65.8%), aged between 25 and 41 years old (47.0%), married (82.4%), of Semai ethnicity (43.0%), obtained secondary education (38.7%) and were not working (67.8%). The median income of the participants was RM500 (IQR = 250, 1000). The characteristics of the participants are displayed in Table 2.

**Table 2:** Socio-demographic characteristics of the study participants

Variables	Frequency (n)	Percentage (%)
Age		
Less than 24 years	77	27.2
25–41 years	133	47.0
42 years and above	73	25.8
Sex		
Men	97	34.2
Women	187	65.8
Ethnicity		
Semai	122	43.0
Jahai	66	23.2
Temiar	96	33.8
Marital status		
Single	40	14.1
Married	234	82.4
Divorced	3	1.1
Widowed	7	2.5
Education level		
Illiterate & Informal	90	31.7
Primary	84	29.6
Secondary and above	110	38.7
Employment status		
Working	91	32.2
Not working	192	67.8
Know someone infected with malaria		
Yes	11	3.9
No	273	96.1
Tested for malaria previously		
Yes	3	1.1
No	281	98.9

### Prevalence of malaria

None of the 284 participants were positive for malaria.

#### Knowledge on malaria

**Fig. 1:**
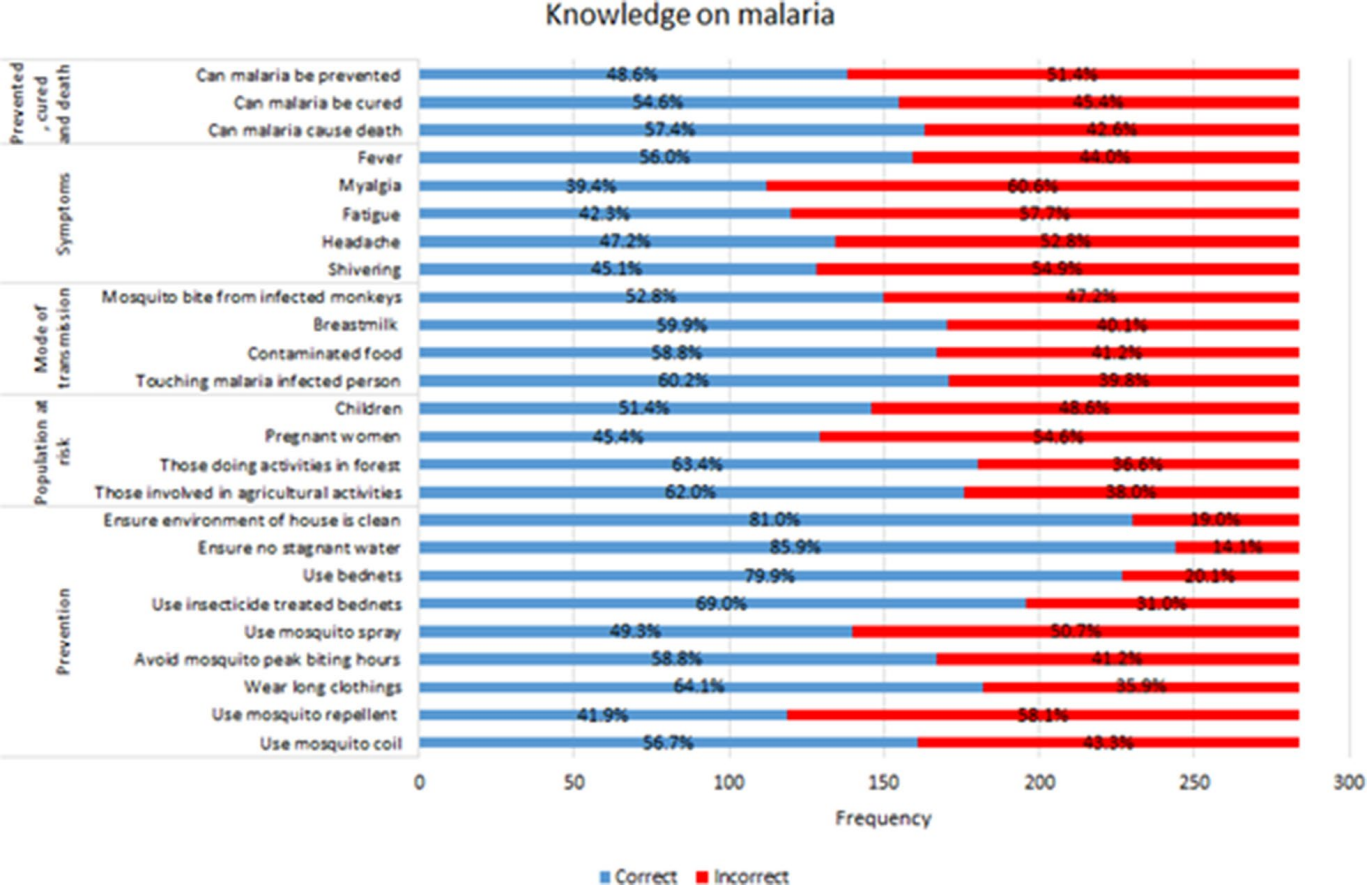
Knowledge on malaria

Figure 1 shows the majority of the participants were aware that by ensuring no stagnant water (85.9%), keeping the house environment clean (81.0%) and usage of bed nets (79.9%) could reduce the population of mosquitoes, thus reduce the risk of contracting malaria. More than half of them were also aware that those doing activities in the forest (63.4%) and involved in agricultural activities (62.0%) were at higher risk for contracting malaria. Only 18.3% of the participants had heard about monkey malaria.

In general, most of the respondents who were interviewed were not sure about monkey malaria but all were aware of malaria in general.

*“I'm not familiar with monkey malaria. I have never heard of it. I've never heard anyone in this area talk about or tell stories about monkey malaria. I only know what malaria is.” (Respondent 6)*

### Attitude towards prevention of human malaria

**Fig. 2:**
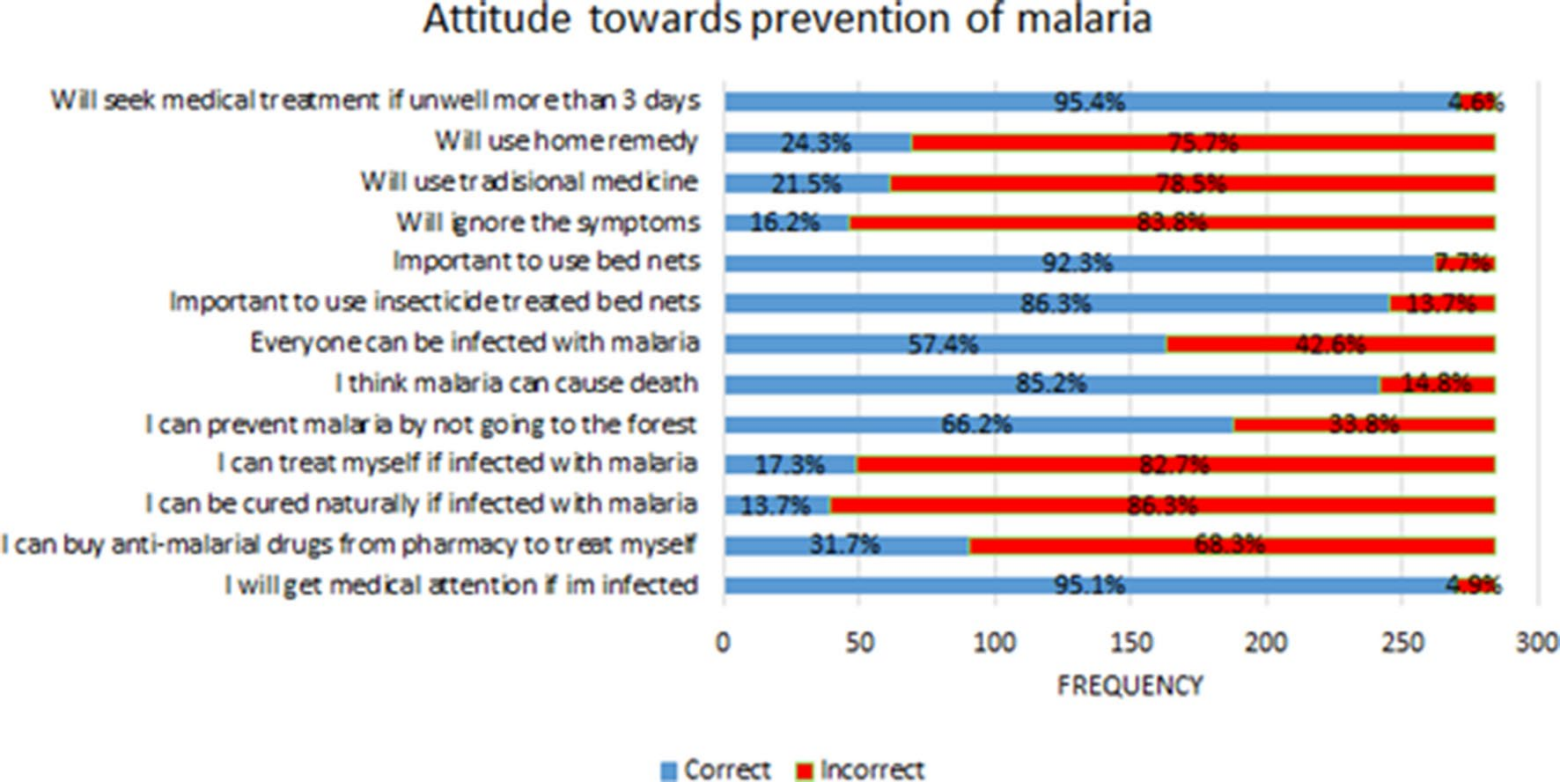
Attitude towards prevention of malaria

Figure 2 shows the attitude towards malaria prevention. In general, majority of the participants have good attitude towards malaria prevention. Most of them will seek medical treatment if they are unwell for more than three days (95.4%), consider it is important to get medical attention if suspected to be infected with malaria (95.1%) and consider it is important to use bed nets when sleeping (92.3%).

During the interviews, their attitude towards treatment was good.

*“We are worried about the mosquito-borne disease. As for me, if my children develop a fever in a single day, I will take them to the doctor right away. I will never wait more than two or three days… we don't have shamans here… we always go to the clinic for treatment… I'll make sure to close all the windows and doors in the evening to keep the mosquitos out of the house” (respondent 6)*

Because malaria is such a debilitating illness, they are concerned about being infected and the resulting health and economic consequences.

*“I'm particularly worried that my children and husband will become infected with malaria. If you get malaria, your body will begin to ache. And when your body isn't healthy, you can't go out to work… my family and I rely on my husband to go out and earn money… if he is sick, we won't have enough money, and no money means no food. If I get sick, who will look after my children? If my neighbour gets it, we are also concerned… because it can spread” (respondent 13)*

### Practices towards prevention of malaria

**Fig. 3:**
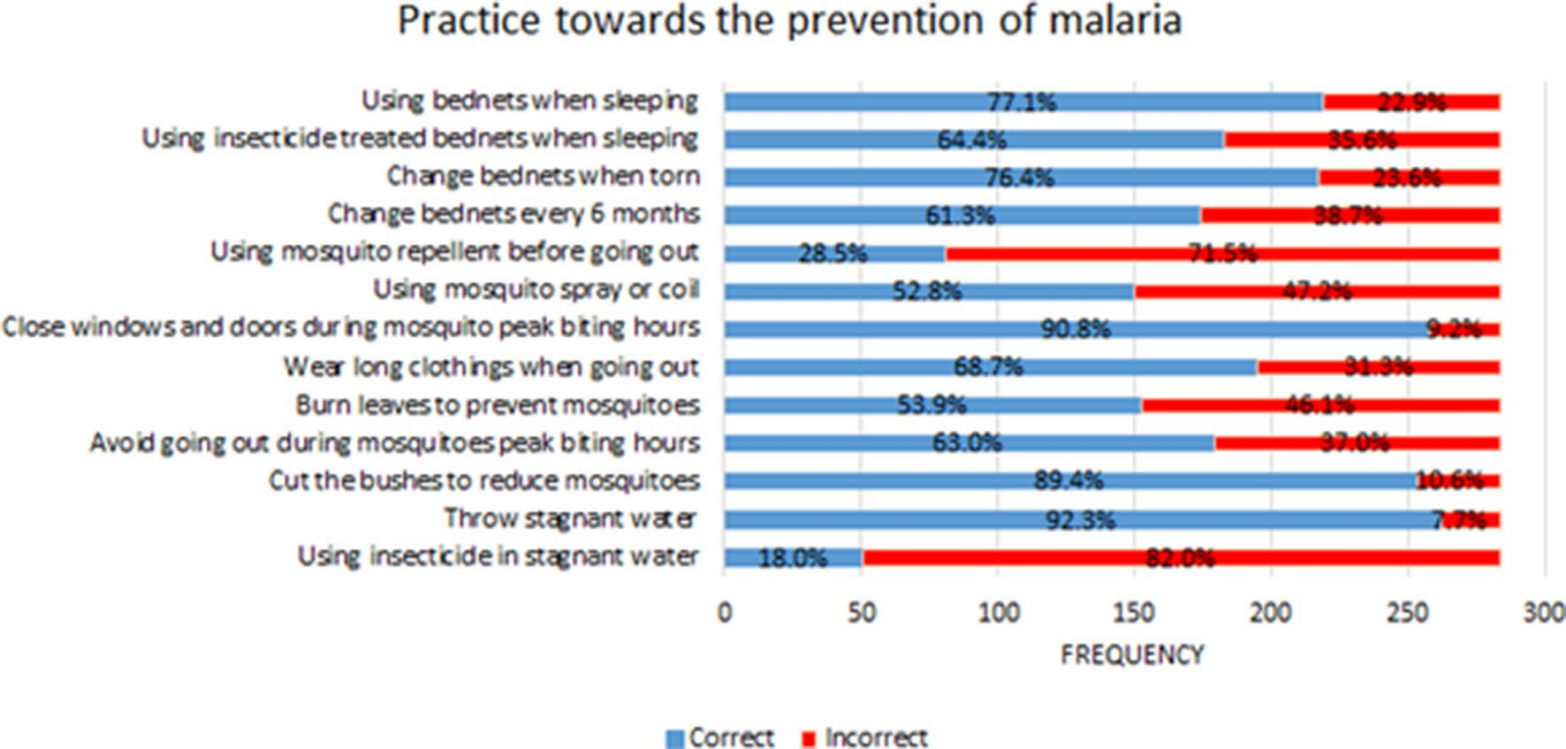
Practices towards prevention of malaria

Figure 3 shows the reported practice towards the prevention of malaria. Majority of the participants reported that they removed stagnant water (92.3%), closed the windows and doors during mosquito peak biting hours (90.8%) and cut the bushes around their house to reduce the mosquitoes breeding areas (89.4%). However, most of them did not use larvicides in stagnant water (82.0%) neither did they use mosquito repellents before going out of their house (71.5%).

Similar findings were elicited during the interviews.

*“To protect my family, I make sure we all use mosquito nets, wear long-sleeved shirts, keep the house clean, dispose of all stagnant water, and clear out the bushes around the house. I do all of this to protect my family and myself from mosquito bites. I'll make certain that no mosquito breeding areas remain. Around 7 p.m., I'll instruct my children to prepare the mosquito net. I've told them (the kids) that once it gets late in the evening, they must close all the windows and doors. I'll clean up the area around the house and light up the dried leaves and trash near the house to get the smoke.”(Respondent 12)*

### Factors associated with knowledge, attitude and practices for the prevention of malaria

Table 3 shows the factors significantly associated with the knowledge, attitude and practice scores. There were statistically significant differences in knowledge and attitude scores among those aged between 25–41, completed secondary education and above, and those who had been tested for malaria previously. There was also significant difference in the knowledge scores among those who knew someone infected with malaria. However, there were no statistically significant difference in the practice scores with all other independent variables.

**Table 3:** Factors significantly associated with the knowledge, attitude and practice scores on prevention of malaria

Knowledge	Mean (SD)	t test or ANOVA (F)	p-value	Post-hoc
Age				
Less than 24 years	13.38 (4.79)	3.649	0.027*	25–41 years > less than 24 years
25–41 years	15.14 (4.62)			
42 years and above	13.89 (5.32)			
Sex				
Men	14.64 (4.70)	0.813	0.417	
Women	14.14 (5.03)			
Ethnicity				
Semai	14.26 (5.00)	1.140	0.321	
Jahai	15.05 (4.88)			
Temiar	13.86 (4.82)			
Marital status				
Married	14.41 (4.86)	− 0.776	0.439	
Single, divorced or widowed	13.82 (5.17)			
Education				
Illiterate & informal	12.33 (5.07)	13.276	< 0.001*	Secondary and above > illiterate & informal education
Primary	14.51 (4.42)			
Secondary and above	15.77 (4.63)			
Employment status				
Working	14.63 (4.91)	− 0.750	0.454	
Not working	14.16 (4.94)			
Monthly Household Income				
< RM 500	14.20 (4.89)	− 1.007	0.317	
≥ RM 500	15.30 (5.09)			
Know someone infected with malaria				
Yes	16.58 (4.02)	− 2.388	0.018*	
No	14.10 (4.94)			
Tested for malaria previously				
Yes	15.18 (4.81)	− 4.978	< 0.001*	
No	12.06 (4.48)			

Attitude	Mean (SD)	t test or ANOVA (F)	p-value	Post-hoc
Age				
Less than 24 years	9.96 (2.38)	4.466	0.012*	25–41 years > less than 24 years
25–41 years	10.82 (1.73)			
42 years and above	10.62 (2.11)			
Sex				
Men	10.53 (1.92)	− 0.035	0.972	
Women	10.53 (2.11)			
Ethnicity				
Semai	10.48 (2.01)	0.065	0.937	
Jahai	10.55 (1.84)			
Temiar	10.58 (2.23)			
Marital status				
Married	10.60 (2.07)	− 1.188	0.236	
Single, divorced or widowed	10.22 (1.94)			
Education				
Illiterate & informal	10.08 (2.46)	3.313	0.038*	Secondary and above > illiterate & informal education
Primary	10.77 (1.59)			
Secondary and above	10.72 (1.94)			
Employment status				
Working	10.75 (1.52)	− 1.431	0.154	
Not working	10.42 (2.25)			
Monthly Household Income				
< RM 500	10.43 (1.55)	− 1.862	0.066	
≥ RM 500	11.00 (1.28)			
Know someone infected with malaria				
Yes	11.25 (1.68)	− 1.805	0.072	
No	10.47 (2.07)			
Tested for malaria previously				
Yes	10.73 (1.87)	− 2.616	0.009*	
No	10.03 (2.39)			

Practice	Mean (SD)	t test or ANOVA (F)	p-value	Post-hoc
Age				
Less than 24 years	8.58 (2.62)	1.822	0.164	
25–41 years	8.50 (2.19)			
42 years and above	7.90 (2.68)			
Sex				
Men	8.06 (2.34)	− 1.512	0.132	
Women	8.52 (2.49)			
Ethnicity				
Semai	8.50 (2.32)	1.943	0.145	
Jahai	8.68 (2.74)			
Temiar	7.98 (2.38)			
Marital status				
Married	8.31 (2.36)	0.777	0.440	
Single, divorced or widowed	8.64 (2.82)			
Education				
Illiterate & informal	8.08 (2.75)	2.309	0.101	
Primary	8.17 (2.03)			
Secondary and above	8.75 (2.45)			
Employment status				
Working	8.37 (2.37)	− 0.029	0.977	
Not working	8.36 (2.49)			
Monthly Household Income				
< RM 500	8.60 (2.49)	0.592	0.556	
≥ RM 500	8.30 (2.23)			
Know someone infected with malaria				
Yes	8.46 (2.59)	− 0.192	0.848	
No	8.36 (2.44)			
Tested for malaria previously				
Yes	8.44 (2.48)	− 0.861	0.390	
No	8.16 (2.37)			

### Correlation between knowledge, attitude and practice scores

Pearson product-moment correlation coefficient revealed significant positive linear correlations between knowledge-attitude (r = 0.346, p < 0.001) and knowledge-practice (r = 0.236, p < 0.001). There was no significant correlation between attitude and practice scores (Table 4).

**Table 4:** Correlation between knowledge, attitude and practice scores

Variable	Correlation coefficient	p-value*
Knowledge–Attitude	0.346	< 0.001
Knowledge–Practice	0.236	< 0.001
Attitude–Practice	0.068	0.257

*Correlation is significant at 0.01 level (2-tailed)

### Factors predicting knowledge and attitude scores

A multiple regression analysis was conducted to determine the combination factors of age, education level, know someone infected with malaria and previously tested for malaria for predicting the knowledge and attitude scores. The results showed that those aged between 25 and 41 years, completed secondary education and above, knowing someone infected with malaria and those who had been tested for malaria previously predicted the knowledge scores F (4, 278) = 12.448, p < 0.001. The adjusted R square was 0.140, which indicates 14% of the variance in the knowledge scores was explained by the model.

The results also showed that those aged between 25 and 41 years and those who had been tested for malaria previously predicted the attitude scores F (3, 279) = 3.957, p = 0.009. The adjusted R square was 0.031, which indicates 3.1% of the variance in the attitude scores was explained by the model (Table 5).

**Table 5:** Multiple linear regression on the predictors of knowledge and attitude scores on malaria

Variables	B	t	p-value	95% CI
Knowledge				
Age (25–41 years)	1.199	2.206	0.028	0.129–2.269
Education level (Secondary and above)	1.949	3.486	0.001	0.848–3.049
Know someone infected with malaria	2.346	2.417	0.016	0.435–4.257
Tested for malaria previously	2.721	4.462	< 0.001	1.520–3.921
Attitude				
Age (25–41 years)	0.494	2.048	0.041	0.019–0.969
Education level (Secondary and above)	0.200	0.807	0.420	− 0.288 to 0.689
Tested for malaria previously	0.643	2.376	0.018	0.110–1.176

## Discussion

This study found no cases of malarial infection in these Orang Asli communities. Those in the middle age group, obtained secondary education, know someone who is infected with malaria and those tested for malaria previously significantly predict the knowledge scores. While those in the middle age group and those tested for malaria previously significantly predicts the attitude scores. Significant correlations were also observed between knowledge-attitude and knowledge-practice. However, no factors significantly predicted practice scores. Similarly, no correlation was observed between attitude and practice.

In general, the malaria rates have decreased worldwide, and Malaysia has made great strides in the reduction of malaria. According to the World Health Organization, Malaysia is currently at the elimination phase. Between the years 2008 and 2017, malaria cases reduced by 98.6% with no malaria cases reported in 2018. However zoonotic malaria cases increased from 376 to 3614 cases [3]. The reduction in human malaria cases is in line with The Malaysia National Malaria Elimination Strategic Plan 2011–2020 with the goal of eliminating local malaria cases. The programme involved strengthening the surveillance system, active and passive case detection, including the biannual routine screening; early case detection, prompt treatment, vector control activities, including the provision of free insecticide-treated bed nets and indoor residual spraying; continuous health education program, training and research [20]. This could explain the findings of the current study.

No case of malaria was detected in this study, similarly with studies conducted in an endemic indigenous locality in Paraguay [21], endemic area of Bashagard District, Iran [22] and in China [23] also showed zero prevalence of human malaria cases. However, there are pockets of cases still reported around Malaysia where Hussin et al. [6] and Ramdzan et al. [24] studies showed prevalence of 16.2% in Peninsular Malaysia and 17.1% of human malaria cases in Sabah among patients attending public health clinic, respectively. Despite the decline in the human malaria incidences [6], it is still a public health threat. In the east Malaysia [25] although low, there is still cases of malaria being reported. Probably due to the differences in geography, movement of people in the forest area, rainfall and vectors.

Due to the extensive spotlight on malaria among these communities by the national malaria elimination programme, the results of this study showed adequate knowledge, attitude and practice in the prevention and control of malaria, which is consistent with other studies [26–30]. Similar to the findings of this study, the study by Munajat et al. conducted among Indigenous population in Kelantan showed almost half of the participants had adequate knowledge on the causes and symptoms of malaria [15]. Similarly, the results were comparable with other international studies which show good knowledge, attitude and practice towards the prevention of malaria [26, 27, 31–33]. Again, these findings suggest the attention given to Malaria has reaped positive outcomes, however the rates of malaria reduction are not equally seen in developing countries worldwide.

Consistent with the literature, the current study found a significant association between age and knowledge scores, whereby those in the middle age group were found to have better knowledge scores than the other counterparts. Similar finding was observed in a study conducted by Aung et al*.* in Myanmar [34]. Higher education levels translates into better understanding of the health promotion materials relates to better knowledge as shown in a study in Cameroon [35] and Nigeria [29]. Similarly higher education levels is associated with better attitude [36]. This could be due to those receiving formal education would have been sensitized and empowered in the school regarding the common diseases, mode of transmission and on prevention of diseases. In a study by Oguntade et al., similar result was observed whereby education level is associated with better attitude towards the prevention of malaria [37]. The same study indicated that the participants who were previously infected with malaria were more aware.

Similar to the current study, a study by Muhammad et al. showed significant association between knowledge and practices on the prevention of malaria [29]. The higher the knowledge that one has, the better that they understand the information that they received and were able to put in practice more efficiently. The current study demonstrates correlation between knowledge and practice. Similar findings was also observed in a study by Shimaponda-Mataa et al. which conducted a study among communities in Zambia [38]. The study showed that those with good knowledge have better practices in the prevention of malaria, especially with the usage of insecticide-treated bed nets. Better knowledge often leads to better practice. With the good level of knowledge and attitude, participants have good practices to avoid from contracting diseases. Knowledge, attitude and practice theory suggest that the acquisition of health-related knowledge, will eventually enhance one’s attitude and leads to good behaviour formation which ultimately will lead to the prevention and control of diseases [39–41]. Activities which involve training and involving the community in recognizing the symptoms of malaria, factors that increase the transmission of malaria and better prevention practices should continue with more novel and targeted approach [42]. However, factors like presence of vectors, suitable environment for the transmission, weather, geographical location and influx of illegal immigrants from malaria endemic countries still pose a heightened the risk malaria. The application of geographic information system mapping (GIS) for the planning of malaria control programme and the emphasis of time-tested malaria prevention and control measures to prevent the occurrence of outbreak in vulnerable and receptive areas [43] are crucial.

## Conclusions

This study shows the effectiveness of the National Malaria Elimination programme; zero prevalence, good knowledge, attitudes and practices relating to malaria control among the indigenous communities. However, other factors like education levels and poverty are equally important to achieve complete malaria elimination in Malaysia. Routine contact between health personnel and the community play an important role in reducing malaria. Improvement in the level of education of these marginalized community will translate into better occupation and income levels which will allow them to have better access to health care and health information. Sustained activities to impart knowledge to these communities will result in better attitude and better preventive practices.

## Strengths and limitations

This study has some strengths and limitations. The main strength of the study is the use of a mixed-method design to gather the data and detailed insights from the participants. Besides that, the study also adopted the usage of gold standard microscopy method for the identification of *Plasmodium* species, rather than the malaria rapid test kit. Nevertheless, there are some limitations that are important to note. Microscopic examination may not accurately diagnose malarial infection and definitive diagnosis only can be made using PCR method. This is true as shown in a study conducted in east Malaysia which showed no infection using microscopic examination but with the use of PCR showed presence of plasmodium parasites [44]. It is important to note that the usage of microscopy has limited sensitivity and it varies according to the experience of the microscopists.

The deployment of cross-sectional study design which does not show any causal relationship between the risk factors and outcome is another limitation. Besides that, the selection of participants were done using a non-probabilistic sampling. Due to the nature of the indigenous people who are semi-nomadic and moves around, it is quite impossible to select them based on random sampling due to unavailability of sampling frame, and nature of their job when most of them goes out to work early during the dawn and are only back during dusk. Due to these limitations, the findings from the study cannot be generalized to the other indigenous population and hence should be interpreted with cautious.

## Recommendations

Further studies should focus on the sub microscopic asymptomatic infections which may remain as reservoir for the transmission of malaria in the local population. Besides that, screening of immigrants prior to the entry into the country as well as periodic screening is essential in minimizing the exposure, incidences and spread of malaria cases, especially with the aim to achieve zero indigenous malaria transmission. Continuous and periodic monitoring not only by the healthcare personnel, but should include the forestry workers, NGOs and those from the acadaemia should be done hand in hand to prevent and control malaria. It is imperative that all the mass media are used to deliver targeted malaria education by disseminating the information to the community, especially tailored according to their level of education. Universal access to preventive measures especially to all high-risk population should also always ubiquitous.

## Data Availability

All data generated or analysed during this study are included in this published article. Nevertheless, datasets are available from the corresponding author on reasonable request.
